# Reactive Scrotal Hydrocele Secondary to Hematoma: A Complication of Laparoscopic Transabdominal Preperitoneal Inguinal Hernioplasty—A Report of Two Cases

**DOI:** 10.1155/cris/7132789

**Published:** 2025-12-11

**Authors:** Goutam Natkunarajah, Alexandros Valorenzos, Thomas Nikolas Valsamidis, Kristian Als Nielsen

**Affiliations:** ^1^ Department of General Surgery, University Hospital of Southern Denmark, Aabenraa, Denmark; ^2^ Department of Emergency Medicine, Aarhus University Hospital, Aarhus, Denmark, auh.dk

**Keywords:** hernia, hydrocele, inguinal hernia, laparoscopy

## Abstract

This case report presents two unique cases of reactive hydrocele formation following transabdominal preperitoneal (TAPP) inguinal hernia repair in adult patients. Although hydrocele development after pediatric inguinal hernia repairs is well‐documented, cases in adults are rare and potentially underreported, likely due to the separation of surgical and urological specialties in adult care. Both patients experienced postoperative hematomas within the inguinal region, which resolved initially but subsequently led to symptomatic hydrocele formation over several months. The first patient, a 65‐year‐old male, developed a left‐sided hydrocele with moderate to severe scrotal pain 8 months postoperatively. The second patient, a 70‐year‐old male, presented with a right‐sided hydrocele 4 months after TAPP repair, initially managed conservatively but later requiring further evaluation due to persistent scrotal swelling and tenderness. Both patients underwent ultrasound imaging, which confirmed the presence of fluid collections around the affected testicles, with no abnormalities in testicular blood flow or echogenicity. A conservative management approach was taken in both cases, and symptoms gradually resolved without surgical intervention. These cases illustrate the potential for postoperative hematomas to exert pressure on lymphatic vessels, possibly leading to reactive hydrocele formation. The delayed onset and symptomatology underscore the importance of recognizing hydrocele as a potential delayed complication of laparoscopic inguinal hernia repair in adults. These cases contribute to the limited literature on this topic and suggest the need for larger cohort studies to explore the pathogenesis, prevalence, and potential correlation between postoperative hematomas and hydrocele formation. Awareness of this rarely reported but impactful complication may improve postoperative management and patient outcomes following TAPP procedures.

## 1. Introduction

Inguinal hernia repair is among the most frequently performed procedures in general surgery worldwide, with an estimated 27% of men and 3% of women undergoing hernia surgery at some point in their lives [1]. Surgical approaches for inguinal hernia repair encompass both open and minimally invasive methods. In recent years, laparoscopic techniques have gained popularity due to potential advantages, such as reduced postoperative pain [2, 3]. Common laparoscopic approaches include Transabdominal preperitoneal (TAPP) and totally extraperitoneal (TEP) repair, each providing access to the inguinal region but differing in anatomical approach. TAPP involves transabdominal entry into the preperitoneal space, while TEP is performed entirely outside the peritoneal cavity. Both techniques have demonstrated similar outcomes [4].

Regardless the technique for hernia repair, a complication that lacks focus in general surgery literature is the development of a hydrocele—a fluid accumulation within the scrotum that can be congenital, idiopathic, or reactive. Reactive hydroceles may arise in response to factors such as trauma, inflammation, or surgery [5]. While hydrocele formation following inguinal hernia repair is well‐documented in pediatric surgery [6–8], reports of hydrocele formation following inguinal hernia surgery in adults are limited [1, 9]. Such cases are noteworthy as they introduce additional diagnostic and management challenges in adults, often resulting in patient discomfort and an extended recovery period and as they might be underreported.

This case report details two adult patients who developed reactive scrotal hydroceles following laparoscopic TAPP hernioplasty performed at the same surgical facility within a 5‐week interval.

## 2. Case Presentation

### 2.1. Patient 1

A 65‐year‐old male presented to the outpatient clinic with left inguinal pain during daily activities. His medical history included hypertension and hypercholesterolemia, managed with amlodipine and atorvastatin, respectively. He had no prior urological history, and his surgical history included a cholecystectomy performed 3 years earlier. Clinical examination revealed a reducible left‐sided indirect inguinal hernia with no scrotal involvement. The patient underwent TAPP for the left inguinal hernia without immediate complications. Postoperatively, he developed a painful inguinal hematoma, which was managed conservatively, with gradual resolution of the hematoma and associated pain. 8 months post‐surgery, the patient returned with persistent left scrotal pain and increased scrotal size, reporting moderate pain (VAS 4) with episodes of intense pain (VAS 9) on palpation. Physical examination showed notable swelling and tenderness in the left testicle. Ultrasound revealed a fluid collection around the left testicle, ~4 × 1.5 cm, with normal echogenicity and blood flow in both testicles. The patient was referred to urology, and by the time of consultation 1 month later, his pain had subsided, occurring only upon direct contact with the testicle. The urologist concluded that surgical intervention was not indicated.

### 2.2. Patient 2

A 70‐year‐old male presented with right‐sided inguinal pain increasingly affecting his daily activities. He had managed a right‐sided inguinal hernia conservatively for 1.5 years with a hernia belt but recently began experiencing progressive pain. His medical history included ischemic heart disease and hypercholesterolemia, for which he was treated with bisoprolol, aspirin, and rosuvastatin. In 2015, he had undergone a four‐vessel coronary artery bypass graft (CABG) following an acute myocardial infarction. He had no significant surgical or urological history. On examination, he was found to have a reducible right‐sided indirect inguinoscrotal hernia. He subsequently underwent TAPP for hernia repair. Intraoperative findings revealed a large, fibrotic hernia sac with dense adhesions to surrounding inguinal tissues, including the funiculus and scrotal base, containing sections of the small intestine and cecum. Initial mobilization attempts were unsuccessful due to extensive fibrosis, so surgeons opted to incise the sac at the highest feasible level to prevent damage to the vas deferens. A Progrip mesh was placed over the inguinal defect, and the peritoneum was closed with V‐Loc 3‐0 sutures. 2 weeks postoperatively, the patient presented with pain and swelling in the right inguinal area and scrotum. Examination indicated tenderness and induration around the inguinal and scrotal regions. A CT scan identified a sharply defined isodense lesion, measuring 78 × 43 × 45 mm medial to the tunica vaginalis, consistent with a hematoma. Expectant management was chosen, with follow‐up scheduled for 2 months later. By the follow‐up visit, the swelling had largely subsided, and the patient reported no pain, so no further follow‐up was scheduled. However, 4 months postoperatively, he returned to the clinic with progressive scrotal swelling and pain. Examination revealed pronounced swelling and tenderness in the right testicle. Ultrasound showed a fluid collection around the right testicle, ~3 × 1 cm, while both testicles exhibited normal echogenicity and blood flow. At follow‐up 1 month later, the patient reported that pain had resolved, with only mild tenderness on palpation. He was referred to urology, where it was determined that surgical intervention was not necessary.

## 3. Discussion

These cases illustrate the development of hydroceles following TAPP procedures complicated by postoperative hematomas. While hydrocele formation is widely documented in pediatric hernia repairs [6–8], reports in adults are limited. This discrepancy may be because pediatric surgeons often manage both hernia and urological cases, whereas in adults, these conditions are typically handled by separate specialties. Consequently, the incidence in adults may be underreported, as surgeons performing hernia operations may not encounter or manage urological conditions routinely.

A 2023 case report suggests a potential relationship between lymphatic vessel injury and hydrocele formation [10]. This report describes the use of indocyanine green (ICG) fluorescent lymphography during laparoscopic inguinal hernia repair in a 59‐year‐old male, which successfully identified lymphatic vessels and any associated leakage. Intraoperatively, ICG visualized the lymphatic structures, revealing injury to left‐sided lymphatic vessels, possibly due to prior surgery. 9 days post‐surgery, a slight postoperative ultrasonic hydrocele was detected on the left side, suggesting a correlation between lymphatic vessel injury and postoperative hydroceles. While some studies address the development of postoperative hydroceles due to lymphatic vessel injury [5, 10], none have specifically investigated the relationship between postoperative hematomas and the subsequent formation of reactive hydroceles.

In the cases presented, postoperative hematomas in the inguinal region may have gradually evolved into symptomatic hydroceles, causing discomfort and limiting daily activities. One possible pathogenesis mechanism is that the hematoma compressed the lymphatic vessels in the area, producing a gradual effect similar to the acute lymphatic vessel injury described in the aforementioned case report and leading to hydrocele formation. These cases underscore the importance of recognizing hydrocele as a delayed complication following laparoscopic hernia repair in adults, contributing to the limited body of literature on this topic. Larger cohort studies are needed to further investigate the pathogenesis, prevalence, and potential association between inguinal hernia repair and hydrocele development in adults.

## Consent

Informed patient consent was obtained for publication of case details.

## Conflicts of Interest

The authors declare no conflicts of interest.

## Funding

This research received no specific grant from any funding agency in the public, commercial, or not‐for profit sectors.

## Data Availability

The data that support the findings of this study are available on request from the corresponding author. The data are not publicly available due to privacy or ethical restrictions.
